# Regular Fat and Reduced Fat Dairy Products Show Similar Associations with Markers of Adolescent Cardiometabolic Health

**DOI:** 10.3390/nu8010022

**Published:** 2016-01-02

**Authors:** Therese A. O’Sullivan, Alexandra P. Bremner, Trevor A. Mori, Lawrence J. Beilin, Charlotte Wilson, Katherine Hafekost, Gina L. Ambrosini, Rae Chi Huang, Wendy H. Oddy

**Affiliations:** 1 School of Exercise and Health Science, Edith Cowan University, 270 Joondalup Dr, Joondalup WA 6027, Australia; cwilso15@our.ecu.edu.au; 2 School of Population Health, The University of Western Australia, Crawley WA 6009, Australia; alexandra.bremner@uwa.edu.au (A.P.B.); gina.ambrosini@uwa.edu.au (G.L.A.); 3 School of Medicine and Pharmacology, The University of Western Australia, Crawley WA 6009, Australia; trevor.mori@uwa.edu.au (T.A.M.); lawrie.beilin@uwa.edu.au (L.J.B.); rae-chi.huang@uwa.edu.au (R.C.H.); 4 Telethon Kids Institute, The University of Western Australia, West Perth WA 6008, Australia; kate.hafekost@westnet.com.au (K.H.); wendyo@ichr.uwa.edu.au (W.H.O.)

**Keywords:** dairy, regular fat, reduced fat, low fat, saturated fat, dairy fat, metabolic, adolescent, blood pressure, cholesterol, Raine study

## Abstract

Reduced fat dairy products are generally recommended for adults and children over the age of two years. However, emerging evidence suggests that dairy fat may not have detrimental health effects. We aimed to investigate prospective associations between consumption of regular *versus* reduced fat dairy products and cardiometabolic risk factors from early to late adolescence. In the West Australian Raine Study, dairy intake was assessed using semi-quantitative food frequency questionnaires in 860 adolescents at 14 and 17-year follow-ups; 582 of these also had blood biochemistry at both points. Using generalized estimating equations, we examined associations with cardiometabolic risk factors. Models incorporated reduced fat and regular fat dairy together (in serves/day) and were adjusted for a range of factors including overall dietary pattern. In boys, there was a mean reduction in diastolic blood pressure of 0.66 mmHg (95% CI 0.23–1.09) per serve of reduced fat dairy and an independent, additional reduction of 0.47 mmHg (95% CI 0.04–0.90) per serve of regular fat dairy. Each additional serve of reduced fat dairy was associated with a 2% reduction in HDL-cholesterol (95% CI 0.97–0.995) and a 2% increase in total: HDL-cholesterol ratio (95% CI 1.002–1.03); these associations were not observed with regular fat products. In girls, there were no significant independent associations observed in fully adjusted models. Although regular fat dairy was associated with a slightly better cholesterol profile in boys, overall, intakes of both regular fat and reduced fat dairy products were associated with similar cardiometabolic associations in adolescents.

## 1. Introduction

Approximately 60%–70% of fat in dairy products such as milk, yoghurt and cheese is saturated fat. Dairy fat is also a source of fat-soluble vitamins and unsaturated fats, including omega-3s. To reduce intakes of saturated fat, dietary guidelines typically recommend choosing mostly reduced fat, rather than regular fat (also known as whole or full fat) dairy products from early childhood [1,2,3]. These guidelines are generally thought to be based on two presumptions: firstly, that the lower energy content of reduced fat products decreases risk of excessive weight gain; and secondly, that the lower saturated fat content decreases risk of cardiometabolic disease [4].

Emerging evidence suggests that despite being high in saturated fat, regular fat dairy products may not contribute to development of obesity or cardiometabolic disease. A systematic review of dairy consumption and obesity in prospective adult cohort studies found that reduced fat dairy products were no more beneficial for weight status than regular fat dairy products, with some studies suggesting that the reverse may be true [5,6]. Similarly, inverse associations with regular fat but not reduced fat dairy and adiposity measures have been reported in children [7,8]. There is also little evidence that higher intakes of regular fat dairy foods increases risk of mortality [9] or chronic disease risk, including metabolic syndrome [10,11,12]. A systematic review of observational studies published in 2013 concluded that existing evidence did not support the hypothesis that dairy fat or regular fat dairy foods contribute to obesity or cardiometabolic disease risk [13].

Few studies have investigated associations between dairy intake, particularly regular compared with reduced fat dairy, and cardiometabolic risk factors in adolescence. Despite adolescence being a critical period of growth and development [14], we have previously reported that intake of dairy products tends to decrease through this period [15]. This may be due in part to avoidance of regular fat dairy products. Evidence suggests that Australian adolescents are failing to consume the 3.5 serves of dairy recommended daily to meet calcium requirements [1,15,16]. If research shows health effects are similar between regular fat and reduced fat dairy products, the enhanced palatability of regular fat products may potentially improve intakes.

We aimed to investigate the association between cardiometabolic risk factors and regular and reduced fat dairy intake from early to late adolescence. We hypothesized that intake of regular fat dairy products would not be associated with increased cardiometabolic risk compared with reduced fat dairy products.

## 2. Subjects and Methods

### 2.1. Subjects

Participants were adolescents in the Western Australian Pregnancy Cohort (Raine) Study. This population-based cohort enrolled 2900 women at 18 weeks gestation between 1989–1991, with 2868 live-born babies followed up at regular intervals [17]. In this study, we report pooled data from 14-year (2003–2006) and 17-year (2006–2009) follow-ups. Ethics committees at King Edward Memorial Hospital and Princess Margaret Hospital for Children approved the research, and adolescents and their parents or guardians gave informed written consent to participate.

### 2.2. Dietary Intake Assessment

Dietary data were collected at the 14-year (*n* = 1631) and 17-year (*n* = 1009) follow-ups using a 212-item semi-quantitative food frequency questionnaire (FFQ) developed by the Commonwealth Scientific and Industrial Research Organization (CSIRO) [18], as previously detailed [19]. The FFQ was based on an Australian food composition database [20] and modified to include snacks and beverages typically eaten by adolescents. It has been shown to reasonably rank intakes when validated against a 3-day food diary in a sub-group of this same cohort [21]. Completed FFQs were checked by a research nurse and forwarded to CSIRO for data entry and verification. We used serves/day of dairy as a measurement unit rather than grams/day, due to inherent differences across the range of dairy products consumed—for example, 40 g of cheese is nutritionally different to 40 g of milk. Based on the Australian Guide to Healthy Eating [22], we calculated total serves based on 300 mg calcium/serve (range 252–350 mg) [15]—for example, one serve of cheese is 40 g, whereas one serve of milk is 250 mL. In addition to dairy products specified in the FFQ (including butter, drinks, milk on cereal, cheese, cream, yoghurt, ice cream, and custard), contributions to milk, butter and cheese categories were extracted from mixed dishes, such as milk puddings, mornay dishes and pizza. To estimate dairy product contributions from these mixed dishes, recipes were standardised against “Cookery the Australian Way” [23] and the “Taste Australia” website [24], and entered into FoodWorks^®^ Professional 2009 dietary software (Xyris Software Pty Ltd, Brisbane, Australia). Dairy content was then determined and added to the applicable category. Dairy products were categorised as either regular or reduced fat based on categories specified in the FFQ, or through searching brand names when specified using FoodWorks analysis or nutrient information panels. Fat content was then compared for that product with industry standards to determine whether it was regular or reduced fat (see the footnote of Table 1). Some non-core dairy products, such as butter, are often not included in studies of dairy intake. However, we chose to include all sources of dairy, particularly high fat sources, because evidence suggests that fat from dairy products, including butter, may have different health effects to other foods high in saturated fat [9].

**Table 1:** Population characteristics, dietary intakes and cardiometabolic factors of Raine Study adolescents at 14- and 17-year follow-ups.

Mean ± SD or *n* (%)	Girls (*n* = 461)		*p*	Boys (*n* = 399)		*p*
	Follow-up Year			Follow-up Year		
	14	17		14	17	
Age (yr)	14.0 ± 0.2	17.0 ± 0.2	<0.001	14.0 ± 0.2	16.9 ± 0.2	<0.001
Height (m)	1.62 ± 0.10	1.66 ± 0.06	<0.001	1.67 ± 0.09	1.79 ± 0.07	<0.001
Weight (kg)	56.3 ± 11.6	63.0 ± 13.0	<0.001	57.8 ± 13.8	71.9 ± 14.4	<0.001
Waist to height ratio	0.45 ± 0.06	0.46 ± 0.06	<0.001	0.453 ± 0.062	0.448 ± 0.057	0.005
BMI (kg/m^2^)	21.3 ± 4.00	22.9 ± 4.31	<0.001	20.6 ± 3.87	22.4 ± 3.92	<0.001
Aerobic fitness ^a^	98.6 ± 19.5	102.3 ± 25.5	0.002	126.4 ± 31.3	158.3 ± 40.7	<0.001
**Family’s characteristics**						
Maternal age (yr)	29.4 ± 5.7		NA	30.0 ± 5.1		NA
Single parent family	83 (18.0)	98 (21.3)	<0.001	51 (12.8)	72 (18.0)	<0.001
Annual family income ($AUD)						
Up to 35,000	100 (21.7)	60 (13.0)	<0.001	71 (17.8)	46 (11.5)	<0.001
35,001–70,000	165 (35.8)	107 (23.2)		138 (34.6)	90 (22.6)	
70,001 and over	189 (41.0)	253 (54.9)		183 (45.9)	247 (61.9)	
**Dietary variables**						
Ever breastfed (yes)	410 (88.9)		NA	353 (88.5)		NA
Energy (MJ/day)	8.75 ± 2.77	8.00 ± 2.64	<0.001	10.18 ± 2.84	10.90 ± 3.46	<0.001
Total dairy (g/day)	439 ± 279 (median 394)	350 ± 247 (median 303)	<0.001	621 ± 350 (median 567)	570 ± 372 (median 495)	0.009
Regular fat dairy (g/day)	228 ± 228 (median 132)	153 ± 194 (median 70)	<0.001	358 ± 373 (median 205)	314 ± 337 (median 160)	0.007
Reduced fat dairy ^b^ (g/day)	211 ± 266 (median 104)	197 ± 218 (median 125)	0.24	263 ± 301 (median 130)	257 ± 321 (median 130)	0.70
Total dairy serves (calcium eq serves/day)	2.18 ± 1.24 (median 1.99)	1.85 ± 1.24 (median 1.63)	<0.001	2.92 ± 1.53 (median 2.67)	2.80 ± 1.71 (median 2.45)	0.18
Regular fat dairy serves (calcium eq serves/day)	1.14 ± 1.05 (median 0.78)	0.84 ± 1.04 (median 0.42)	<0.001	1.69 ± 1.70 (median 1.06)	1.55 ± 1.58 (median 1.00)	0.06
Reduced fat dairy serves (calcium eq serves/day)	1.04 ± 1.24 (median 0.54)	1.01 ± 1.06 (median 0.73)	0.56	1.23 ± 1.35 (median 0.65)	1.25 ± 1.49 (median 0.63)	0.70
Calcium (mg/day)	1033 ± 480 (median 938)	913 ± 465 (median 855)	<0.001	1295 ± 541 (median 1226)	1291 ± 656 (median 1174)	0.90
Protein (g/day)	85.5 ± 27.3	79.2 ± 28.8	<0.001	101 ± 28.6	107 ± 38.0	0.002
Carbohydrate (g/day)	253 ± 87	228 ± 76	<0.001	292 ± 86	305 ± 102	0.02
Total fat (g/day)	82.7 ± 29.6	70.8 ± 28.7	<0.001	96.3 ± 32.8	99.4 ± 38.5	0.12
Polyunsat fat (g/day)	12.9 ± 6.12	10.4 ± 5.67	<0.001	14.2 ± 6.89	13.3 ± 7.07	0.04
Monounsat fat (g/day)	28.5 ± 10.3	24.1 ± 9.74	<0.001	33.3 ± 11.7	34.0 ± 13.2	0.31
Saturated fat (g/day)	35.8 ± 14.5	30.7 ± 14.4	<0.001	42.7 ± 16.6	44.6 ± 19.6	0.05
Healthy diet pattern score ^c^	0.05 ± 0.85 (median −0.10)	0.06 ± 0.88 (median −0.08)	0.76	−0.01 ± 0.88 (median −0.38)	−0.07 ± 0.89 (median −0.41)	0.14
Western diet pattern score ^c^	−0.21 ± 0.83 (median −0.07)	−0.32 ± 0.68 (median −0.21)	0.005	0.07 ± 0.81 (median −0.09)	0.35 ± 0.90 (median 0.23)	<0.001
Reporting status						
Under-reporter	161 (34.9)	221 (47.9)	<0.001	46 (11.5)	65 (18.5)	0.03
Plausible-reporter	237 (51.4)	171 (37.1)		256 (64.2)	228 (65.0)	
Over-reporter	14 (3.0)	7 (1.5)		60 (15.0)	58 (16.5)	
**Cardiometabolic factors**						
Diastolic blood pressure (mmHg)	59 ± 7	60 ± 6	0.10	59 ± 7	59 ± 7	0.33
Systolic blood pressure (mmHg)	109 ± 9	110 ± 10	0.14	114 ± 11	119 ± 10	<0.001
HDL-cholesterol (mmol/L)	1.44 ± 0.32	1.42 ± 0.31	0.19	1.37 ± 0.29	1.22 ± 0.24	<0.001
LDL-cholesterol (mmol/L)	2.36 ± 0.59	2.39 ± 0.61	0.36	2.26 ± 0.67	2.22 ± 0.67	0.15
Total cholesterol (mmol/L)	4.27 ± 0.63	4.26 ± 0.71	0.85	4.05 ± 0.73	3.91 ± 0.74	<0.001
Total:HDL-cholesterol ratio	3.11 ± 0.81	3.12 ± 0.71	0.80	3.05 ± 0.74	3.32 ± 0.84	<0.001
Triglycerides (mmol/L)	1.02 ± 0.41	1.00 ± 0.42	0.36	0.91 ± 0.45	1.03 ± 0.57	0.001
Glucose (mmol/L)	4.68 ± 0.39	4.62 ± 0.92	0.011	4.86 ± 0.38	4.78 ± 0.49	0.002
Insulin (mU/L)	11.9 ± 6.66 (median 10.5)	9.77 ± 14.4 (median 8.34)	0.003	11.1 ± 8.12 (median 9.50)	8.57 ± 6.62 (median 7.16)	<0.001
HOMA-IR ^d^	2.50 ± 1.57 (median 2.18)	2.07 ± 3.60 (median 1.69)	0.025	2.45 ± 2.15 (median 2.01)	1.85 ± 1.53 (median 1.51)	<0.001
CRP (mg/L)	1.68 ± 5.27 (median 0.48)	2.39 ± 4.48 (median 0.66)	0.12	1.24 ± 1.99 (median 0.52)	1.31 ± 1.96 (median 0.40)	0.72
MetS IDF ^e^ (yes)	20 (4.3)	15 (3.3)	>0.99	11 (2.8)	9 (2.3)	>0.99
Cohort MetS cluster ^f^ (high risk)	103 (22.3)	59 (12.8)	<0.001	64 (16.0)	39 (9.8)	0.004

Percentages may not add to 100% if values were missing; *p*-values for paired *t*-tests or McNemar tests; NA Test not applicable, or test not valid due to insufficient data; AUD = Australian dollars; BMI = body mass index; CRP = C-reactive protein; LDL = low density lipoprotein; HDL = high density lipoprotein; ^a^ The physical working capacity test at a heart rate of 170 beats per minute (PWC170) was used to estimate aerobic fitness; ^b^ Reduced fat classifications: milk < 3 g fat/100 mL, cheese < 16 g fat/100 g, butter < 50 g fat/100 g, ice-cream < 7 g fat/100 g, yoghurt < 3 g fat/100 g, dairy dessert/custard < 3g fat/100 g, cream < 30 g fat/100 g; ^c^ Dietary patterns previously identified in the Raine cohort that can be considered to be a measure of overall dietary quality—the healthy pattern is characterized by high intakes of fiber and micronutrients, while the Western dietary pattern is characterized by high intakes of fat, saturated fat, cholesterol and refined sugars [19]; ^d^ Homeostasis model assessment for insulin resistance (HOMA-IR) [25,26]; ^e^ Metabolic syndrome as defined by the International Diabetes Federation (IDF) paediatric criteria [27]; ^f^ Cohort based categorization into “high or low metabolic syndrome risk” groups derived using cluster analysis [28].

Further dietary aspects considered from the FFQ for analyses in this study were total daily energy intake and two major dietary patterns previously identified in the Raine cohort that can be considered to be an estimate of overall dietary quality. These patterns, defined as “healthy” and “Western”, were identified at the 14- and 17-year follow-ups through factor analysis [19]. The healthy pattern is characterized by high intakes of fiber and micronutrients, whereas the Western is characterized by high intakes of fat, saturated fat, cholesterol and refined sugars. As adolescents who consume reduced fat dairy may differ in other dietary habits to those who consume regular fat dairy, this additional information assisted to partially account for these differences. In addition, the ratio of reported energy intake relative to the estimated energy requirement was used to classify potential under, plausible or over-reporters based on the Goldberg method, as previously described [29]. Rather than excluding possible under or over-reporters, this categorical variable was considered as a potential confounder in statistical models [30].

### 2.3. Metabolic Syndrome and Cardiometabolic Risk Factor Assessments

We chose to use both the International Diabetes Federation (IDF) paediatric criteria [27], as recommended by the American Heart Association [31], and a cohort based categorization into “high or low metabolic risk” groups derived using cluster analysis [28] to define metabolic syndrome (MetS). IDF criteria require a high waist circumference in addition to at least two of the following: high fasting serum triglycerides; low fasting serum high-density lipoprotein (HDL) cholesterol; high systolic or diastolic blood pressure (BP); or high fasting plasma glucose concentrations. Thresholds for high or low subgroups vary by gender and age as published previously [27]. The two-step cluster analysis utilized anthropometric, BP, and lipid assessments, along with the homeostasis model assessment for insulin resistance (HOMA-IR) [25,26] to determine natural groupings in the data and maximize within-group similarities and between-group differences. Analyses were performed separately by gender [28], with adolescents grouped into either a high-risk metabolic cluster or a low-risk metabolic cluster. At both 14- and 17-years, membership of the high-risk cluster was significantly associated with increased systolic and diastolic BP, waist circumference, fasting insulin, glucose and HOMA-IR, fasting triglycerides, and low-density lipoprotein (LDL) cholesterol, and lower HDL-cholesterol compared with adolescents in the low-risk metabolic cluster [32].

Trained researchers measured waist circumference to the nearest 0.1 cm at the level of the umbilicus until two readings were within a centimetre of each other. Weight was measured to the nearest 100 g using a Wedderburn Digital Chair Scale, and height to the nearest 0.1 cm using a Holtain Stadiometer. Body mass index (BMI) was calculated as weight in kilograms divided by height in metres squared. Waist to height ratio was used as an indicator of abdominal obesity [33].

BP measurements were taken after a five-minute rest using a Dinamap ProCare 100 automatic oscillometric recorder (GE Healthcare Technologies, Rydalmere, Australia). The appropriate cuff for body size was used and placed on the right arm. BP readings were obtained in the seated position at 14 years and in the supine position at 17 years. Six measurements were taken over 10 min; the first was disregarded and the mean of the next five measurements was calculated.

Phlebotomists visited adolescents at home early in the morning to obtain fasting blood samples. PathWest Laboratories at Royal Perth Hospital conducted the assays [28]. Serum total cholesterol and triglycerides were determined enzymatically on the Cobas MIRA analyser (Roche Diagnostics, Basel, Switzerland) with reagents from Trace Scientific (Melbourne, Australia). HDL cholesterol was determined on a heparin-manganese supernatant. Serum glucose was measured with an automated Technicon Axon Analyzer (Bayer Diagnostics, Sydney, Australia) by using a hexokinase method. Serum insulin was measured by radioimmunoassay with an automated immunoassay analyzer (Tosoh Corporation, Tokyo, Japan). Glucose and insulin values were used to derive HOMA-IR, a measure of insulin resistance [25,26]. C-reactive protein (CRP), a marker of inflammation in the body, was assessed using a high-sensitivity monoclonal antibody assay (Dade Behring Marburg, Marburg, Germany).

### 2.4. Additional Assessments

The primary caregiver provided sociodemographic information including family structure, family income, family medical history, parental age and employment at the 14- and 17-year follow-ups. The mother or primary caregiver also provided information on early infant feeding, including breastfeeding and solids, during the first years of life. The physical working capacity test at a heart rate of 170 beats per minute (PWC170) was used to estimate aerobic fitness at both time points. This test has been shown to be a valid predictor of VO2max in adolescents [34].

### 2.5. Statistical Analysis

Subjects with extreme daily energy intakes were excluded (<3000 or >20000 kJ/day, as previously used in adolescent studies [21,35]. Baseline characteristics were summarized using means and standard deviations (SDs) for continuous measures and percentages for categorical variables. Medians were included for markedly skewed measures. Paired *t*-tests and McNemar tests were used to determine differences in the characteristics between the 14-and 17-year follow-ups. Paired *t*-tests were also used to evaluate differences in regular and reduced fat dairy intake in groups classified as high or low metabolic risk.

Generalized estimating equations (GEEs) were used to examine associations between dietary intakes with the MetS as defined by IDF adolescent specific criteria and the cohort metabolic cluster, and related metabolic factors. By taking correlation into account, GEEs allowed the data from both follow-ups to be used [36]. Potential confounding factors were investigated for inclusion in models depending on statistical significance, model fit and colinearity. Regular fat dairy (serves/day) and reduced fat dairy (serves/day) were considered together as explanatory variables in models that were initially adjusted for age, total daily energy intake and dietary misreporting status. Regular and reduced fat dairy were included together to account for the likelihood that some adolescents would have a lower regular fat dairy intake due to consuming predominantly reduced fat dairy, and *vice-versa* (correlation between regular and reduced fat dairy was −0.45 at 14 years and −0.31 at 17 years). Dietary intake of total dairy (serves/day) was also considered in separate models. All models were further adjusted for aerobic fitness, maternal age, ever breastfed (yes/no), and healthy and Western dietary pattern *z*-scores. Models of CRP, HOMA-IR, LDL-cholesterol, HDL-cholesterol, blood pressure and the ratio of total to HDL cholesterol were additionally adjusted for BMI.

For the binary response variables IDF and cohort cluster MetS, the models’ odds ratios (ORs), and 95% confidence intervals (CIs), estimate the factor change in odds of having the syndrome associated with an increase in consumption of one serve of dairy. Variables that were not normally distributed, such as waist-height ratio, CRP, HOMA-IR, HDL-cholesterol, LDL-cholesterol and the total: HDL-cholesterol ratio were log transformed and model estimates were interpreted as the percentage (factor) change in each measure associated with an increase in consumption of one serve of dairy. Systolic and diastolic BPs were not skewed, and model coefficients (B) provide the mean change in pressure with a one serve increase in consumption. Data were analysed using IBM SPSS Statistics (Version 21.0) [37] and statistical significance was set at 5%. No mathematical correction was made for testing multiple associations, instead all results including 95% CIs, and *p*-values < 0.05 are reported.

## 3. Results

**Figure 1:**
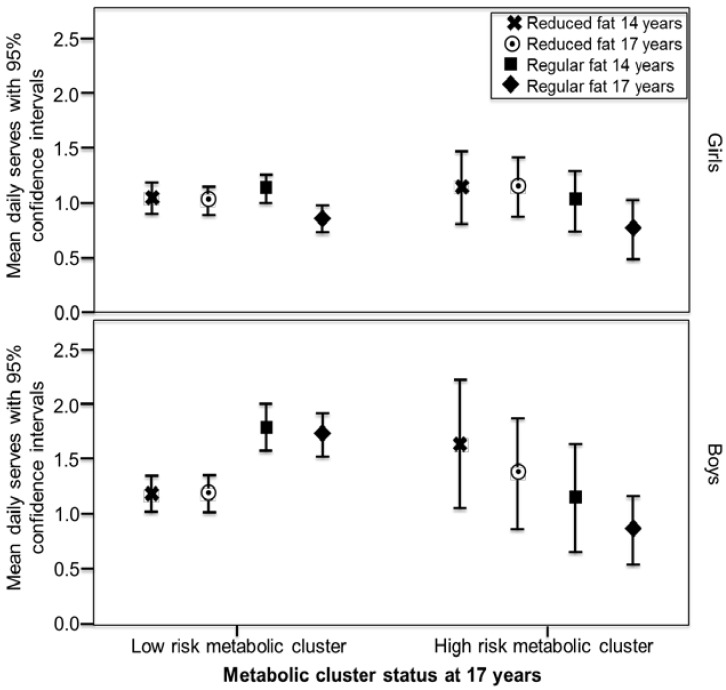
Mean intake of reduced fat and regular fat dairy (serves/day) in girls and boys over the 14 and 17-year follow-ups, according to metabolic cluster status at the 17-year follow-up. In boys in the low-risk metabolic cluster, mean intakes of regular fat products at both 14 and 17 years were significantly higher than intakes of reduced fat products (paired *t*-tests, *p* < 0.001 and *p* = 0.001, respectively). ^a^ Reduced fat classifications: milk < 3 g fat/100 mL, cheese < 16 g fat/100 g, butter < 50 g fat/ 100 g, ice-cream < 7 g fat/100 g, yoghurt < 3 g fat/100 g, dairy dessert/custard < 3 g fat/ 100 g, cream < 30 g fat/100 g; ^b^ Classification as high or low metabolic risk based on cluster analysis in the cohort [28].

**Figure 2:**
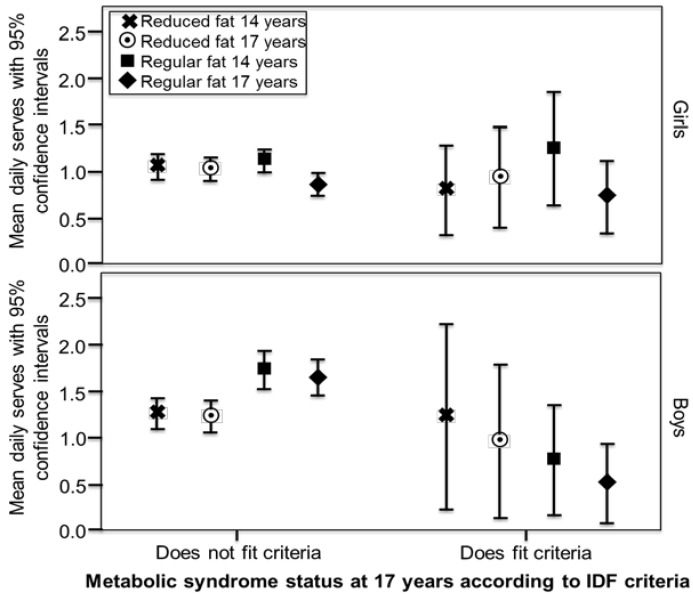
Mean intake of reduced fata and regular fat dairy (serves/day) in girls and boys over the 14 and 17-year follow-ups, according to metabolic syndrome status at the 17 year follow up. In boys who did not fit the metabolic syndrome criteria, mean intakes of regular fat products at both 14 and 17 years were significantly higher than intakes of reduced fat products (paired *t*-tests, *p* = 0.005 and *p* = 0.008, respectively). ^a^ Reduced fat classifications: milk < 3 g fat/100 mL, cheese < 16 g fat/100 g, butter < 50 g fat/ 100 g, ice-cream < 7 g fat/100 g, yoghurt < 3 g fat/100 g, dairy dessert/custard < 3 g fat/ 100 g, cream < 30 g fat/100 g; ^b^ According to the International Diabetes Federation (IDF) adolescent specific criteria [27].

FFQs were completed by 1631 adolescents at the 14-year follow-up and 1009 at 17-years, with 860 (46.4% male) providing usable dietary data from both follow-ups. Of these, 582 also had blood biochemistry at both follow-ups, although more had physical measures or at least one blood assessment. Characteristics, dietary intakes and cardiometabolic variables are reported in Table 1. Detailed information on dairy intake for this cohort has been published previously [15]. Differences in Raine Study adolescents participating in dietary assessment have been previously reported to be higher family income, older mothers, and a lower BMI than those adolescents who chose not to participate in this aspect of the longitudinal study [21,38,39]. Mean intakes of low and regular fat dairy by MetS status are displayed in Figure 1 and Figure 2. In boys in the low-risk metabolic cluster, mean intakes of regular fat products at both 14 and 17 years were significantly higher than intakes of reduced fat products (paired *t*-tests, *p* < 0.001 and *p* = 0.001, respectively). Results were similar for boys who did not have MetS as defined by IDF criteria (*p* = 0.005 and *p* = 0.008, respectively). In boys in the high-risk metabolic cluster and those with MetS as defined by IDF criteria, mean intakes of regular fat products at 14 and 17 years were lower than intakes of reduced fat products, but the differences were not significant. Intakes in girls were similar between MetS risk groups, with regular fat dairy at 17 years showing the lowest level of intake in both high and low risk groups.

GEE model estimates are presented in Table 2 and Table 3. Although there was a 2% reduction in the total: HDL-cholesterol ratio for each serve of total dairy intake in girls (95% CI 0.97–0.99; *p* = 0.038) in the model adjusted for age, kilojoule intake and reporting bias, the reduction was not significant in the fully adjusted model. In boys, after full adjustment, there was a decrease in mean diastolic BP of 0.54 mmHg (95% CI 0.17–0.92; *p* = 0.004) along with a decrease in HDL-cholesterol of 1% (95% CI 0.98–0.998; *p* = 0.008) per total dairy serve (Table 2).

Table 3 presents the explanatory effects when total dairy intake was divided into reduced fat and regular fat components. Low and regular fat dairy intakes both displayed similar associations with cardiometabolic risk factors. In boys, a significant reduction in diastolic BP was associated with both reduced fat and regular fat dairy consumption. After full adjustment, there was a mean reduction in diastolic BP of 0.66 mmHg (95% CI 0.23–1.09; *p* = 0.002) per serve of reduced fat dairy and an independent, additional reduction of 0.47 mmHg (95% CI 0.04–0.90; p = 0.034) per serve of regular fat dairy. The only other significant association in boys after full adjustment was that for each additional serve of reduced fat dairy there was a 2% reduction in HDL-cholesterol (95% CI 0.97–0.995; *p* = 0.005) and a 2% increase in the total: HDL-cholesterol ratio (95% CI 1.002–1.03; *p* = 0.023); this was not observed with regular fat products. In addition, waist to height ratio was significantly inversely associated with regular fat dairy after partial (OR 0.993, 95%CI 0.988–999, *p* = 0.014) but not full adjustment for potential confounding factors. In girls, the only significant association was that for each additional serve of regular fat dairy there was a 2% decrease in the total: HDL-cholesterol ratio (95% CI 0.97, 0.99; *p* = 0.013) in the model adjusted for age, kilojoule intake and reporting status, but this association did not remain significant after full adjustment. No significant associations were observed with metabolic syndrome and dairy intakes for boys or girls.

**Table 2:** Multivariable explanatory effects of total dairy intake (serves/day) on cardiometabolic risk factors.

Outcomes	Girls Model 1 ^a^	Girls Model 2 ^b^	Boys Model 1	Boys Model 2
	OR ^c^ or B ^d^ (95% CI)	OR or B (95% CI)	OR or B (95% CI)	OR or B (95% CI)
IDF met	0.72 (0.48, 1.08)	0.74 (0.48, 1.13)	0.88 (0.58, 1.35)	0.98 (0.61, 1.58)
Cluster met	0.98 (0.83, 1.15)	0.87 (0.71, 1.07)	1.13 (0.94, 1.35)	1.09 (0.90, 1.33)
Waist: height	1.00 (0.99, 1.00)	1.00 (0.99, 1.01)	1.00 (0.99, 1.00)	1.00 (0.99, 1.01)
CRP	1.00 (0.90, 1.12)	1.05 (0.94, 1.18)	0.99 (0.93, 1.07)	1.02 (0.95, 1.10)
HOMA-IR	0.97 (0.93, 1.02)	1.03 (0.98, 1.08)	0.97 (0.94, 1.01)	0.99 (0.95, 1.03)
HDL	1.01 (0.99, 1.03)	1.01 (0.99, 1.03)	0.99 (0.98, 1.001)	0.99 (0.98, 0.998) *
LDL	0.99 (0.97, 1.00)	0.99 (0.97, 1.01)	1.00 (0.99, 1.02)	1.00 (0.98, 1.01)
Total: HDL	0.98 (0.97, 0.999) *	0.99 (0.97, 1.01)	1.01 (0.99, 1.02)	1.01 (1.00, 1.02
SBP	0.40 (−0.33, 1.14)	0.56 (−0.25, 1.36)	−0.25 (−0.84, 0.35)	−0.45 (−1.04, 0.13)
DBP	−0.13 (−0.76, 0.29)	−0.12 (−0.65, 0.40)	−0.51 (−0.84, −0.19) *	−0.54 (−0.92, −0.17) *

IDF met = classified as having metabolic syndrome according to the International Diabetes Federation (IDF) adolescent specific criteria [27]; Cluster met = classified as being in the high metabolic risk cluster based on cluster analysis in the cohort [28]; CRP = C-reactive protein (mg/L); HOMA-IR = homeostasis model assessment for insulin resistance [25,26]; HDL = high density lipoprotein cholesterol (mmol/L); LDL = low density lipoprotein cholesterol (mmol/L); SBP = systolic blood pressure (mmHg); DBP = diastolic blood pressure (mmHg); * = *p* < 0.05; ^a^ Model 1 adjusted for age, kilojoule intake and reporting status; ^b^ Model 2 further adjusted for aerobic fitness, maternal age, BMI, ever breastfed (yes/no), and healthy and Western dietary pattern scores, except models of MetS IDF, MetS cluster, and waist-height ratio, which did not include adjustment for BMI; ^c^ IDF & cluster met—odds ratio (OR) is the factor change in odds of being classified as having MetS (defined by those criteria) associated with a one serve increase in total dairy consumption; waist-height ratio, CRP, HOMA-IR, HDL- and LDL-cholesterol, and total: HDL cholesterol ratio—percentage (factor) change in each measure associated with a one serve increase in total dairy consumption. The change is statistically significant at the 5% level if the 95% CI excludes one (these response variables were logged for modelling); ^d^ SBP, DBP—coefficient (B) is mean change in pressure with a one serve increase in total dairy consumption. The change is statistically significant at the 5% level if the 95% CI excludes zero.

**Table 3:** Multivariable explanatory effects of reduced and regular fat dairy intakes (serves/day) on cardiometabolic risk factors.

	Outcome	Model	Reduced Fat	Regular Fat
			OR ^c^ or B ^d^ (95% CI)	OR ^e^ or B ^f^ (95% CI)
**Girls**	IDF met	1 ^a^	0.75 (0.49, 1.14)	0.67 (0.39, 1.13)
		2 ^b^	0.79 (0.50, 1.25)	0.66 (0.38, 1.15)
	Cluster met	1	0.97 (0.81, 1.15)	1.00 (0.79, 1.26)
		2	0.84 (0.68, 1.04)	0.95 (0.72, 1.24)
	Waist: height	1	1.00 (0.99, 1.01)	0.99 (0.986, 1.00)
		2	1.00 (0.99, 1.01)	1.00 (0.99, 1.01)
	CRP	1	1.01 (0.91, 1.13)	0.97 (0.84, 1.12)
		2	1.08 (0.96, 1.21)	0.98 (0.84, 1.14)
	HOMA-IR	1	0.97 (0.92, 1.02)	0.98 (0.92, 1.03)
		2	1.02 (0.97, 1.07)	1.04 (0.99, 1.10)
	HDL	1	1.01 (0.99, 1.03)	1.02 (0.99, 1.05)
		2	1.00 (0.98, 1.02)	1.01 (0.99, 1.04)
	LDL	1	0.98 (0.96, 1.00)	0.99 (0.97, 1.02)
		2	0.99 (0.97, 1.01)	0.99 (0.97, 1.02)
	Total: HDL	1	0.98 (0.97, 0.99) *	0.98 (0.96, 1.00)
		2	0.99 (0.97, 1.01)	0.98 (0.96, 1.01)
	SBP	1	0.61 (−0.17, 1.38)	−0.09 (−0.99, 0.81)
		2	0.73 (−0.13, 1.59)	0.20 (−0.77, 1.18)
	DBP	1	−0.14 (−0.61, 0.33)	−0.11 (−0.72, 0.51)
		2	−0.13 (−0.67, 0.41)	−0.11 (−0.79, 0.58)
**Boys**	IDF met	1	1.01 (0.65, 1.57)	0.62 (0.25, 1.51)
		2	1.22 (0.67, 2.21)	0.73 (0.31, 1.67)
	Cluster met	1	1.03 (0.84, 1.25)	1.24 (1.00, 1.55)
		2	0.97 (0.77, 1.22)	1.19 (0.93, 1.51)
	Waist: height	1	1.00 (0.99, 1.01)	0.993 (0.988, 0.999) *
		2	1.01 (1.00, 1.01)	0.996 (0.99, 1.00)
	CRP	1	1.01 (0.94, 1.10)	0.98 (0.91, 1.06)
		2	1.04 (0.96, 1.13)	1.01 (0.94, 1.10)
	HOMA-IR	1	0.97 (0.93, 1.02)	0.97 (0.93, 1.01)
		2	0.98 (0.93, 1.02)	0.99 (0.95, 1.04)
	HDL	1	0.99 (0.98, 0.999) *	1.00 (0.99, 1.01)
		2	0.98 (0.97, 0.995) *	0.99 (0.98, 1.00)
	LDL	1	1.00 (0.98, 1.01)	1.00 (0.99, 1.02)
		2	1.00 (0.98, 1.02)	1.00 (0.98, 1.02)
	Total: HDL	1	1.01 (1.00, 1.02)	1.00 (0.99, 1.01)
		2	1.02 (1.002, 1.03) *	1.01 (1.00, 1.02)
	SBP	1	0.01 (−0.71, 0.74)	−0.43 (−1.07, 1.78)
		2	−0.52 (−1.24, 0.20)	−0.41 (−1.04, 0.22)
	DBP	1	−0.56 (−0.96, −0.17) *	−0.47 (−0.84, −0.10) *
		2	−0.66 (−1.09, −0.23) *	−0.47 (−0.90, −0.04) *

Reduced fat classifications: milk < 3g fat/100 mL, cheese < 16 g fat/100 g, butter < 50 g fat/100 g, ice-cream < 7 g fat/100 g, yoghurt < 3 g fat/100 g, dairy dessert/custard < 3 g fat/100 g, cream < 30 g fat/100 g; IDF met = classified as having metabolic syndrome as defined by the International Diabetes Federation (IDF) paediatric criteria [27]; Cluster met = classified as being in the high metabolic risk cluster based on cluster analysis in the cohort [28]; CRP = C-reactive protein (mg/L); HOMA-IR = homeostasis model assessment for insulin resistance [25,26]; HDL = high density lipoprotein cholesterol (mmol/L); LDL = low density lipoprotein cholesterol (mmol/L); SBP = systolic blood pressure (mmHg); DBP = diastolic blood pressure (mmHg); * *p* < 0.05; Reduced fat and regular fat dairy products (serves/day) are included in the models together; ^a^ Model 1 adjusted for age, kilojoule intake and reporting status; ^b^ Model 2 further adjusted for aerobic fitness, maternal age, BMI, ever breastfed (yes/no), and healthy and Western dietary pattern scores, except models of MetS IDF and MetS cluster which did not include adjustment for BMI; ^c^ IDF & cluster met—odds ratio (OR) is the factor change in odds of being classified as having MetS (defined by those criteria) associated with a one serve increase in reduced fat dairy consumption (adjusted for regular fat dairy consumption); CRP, HOMA-IR, HDL- and LDL-cholesterol, and total: HDL-cholesterol ratio—percentage (factor) change in each measure associated with a one serve increase in reduced fat dairy consumption (adjusted for regular fat dairy consumption). The change is statistically significant at the 5% level if the 95% CI excludes one; ^d^ SBP, DBP—coefficient (B) is mean change in pressure with a one serve increase in reduced fat dairy consumption (adjusted for regular fat dairy consumption). The change is statistically significant at the 5% level if the 95% CI excludes zero; ^e^ IDF & cluster met—odds ratio (OR) is the factor change in odds of being classified as having MetS (defined by those criteria) associated with a one serve increase in regular fat dairy consumption (adjusted for reduced fat dairy consumption); CRP, HOMA-IR, HDL- and LDL-cholesterol, and total: HDL-cholesterol ratio—percentage (factor) change in each measure associated with a one serve increase in regular fat dairy consumption (adjusted for reduced fat dairy consumption). The change is statistically significant at the 5% level if the 95% CI excludes one; ^f^ SBP, DBP—coefficient (B) is mean change in pressure with a one serve increase in regular fat dairy consumption (adjusted for reduced fat dairy consumption). The change is statistically significant at the 5% level if the 95% CI excludes zero.

## 4. Discussion

This study provides a unique contribution to the dairy literature by comparing reduced fat and regular fat product intake with cardiometabolic risk factors, in girls and boys, over the period from early to late adolescence. Adolescence is an important period of growth and development and it is valuable to have investigations specific to this group. Overall, intakes of both regular and reduced fat dairy products during adolescence were similarly associated with cardiometabolic risk factors after adjustment for potential confounding factors. In accordance with our hypothesis, regular fat dairy product intake was not associated with increased cardiometabolic disease risk. Both low and regular fat dairy were inversely associated with diastolic BP in boys, with one serve equating to a small decrease of approximately 0.5 mmHg. In addition, reduced fat dairy intake was inversely associated with HDL-cholesterol, but the association was small. In girls, however, there were no significant associations after potential confounding factors were taken into account.

Boys in the low-risk metabolic groups in late adolescence consumed higher mean intakes of regular fat than reduced fat dairy products in early and late adolescence. Likewise, in the high-risk metabolic groups in late adolescence, both boys and girls consumed less regular fat dairy compared to reduced fat. However, there were no significant differences when modelled with potential confounding factors.

The beneficial association we observed with dairy and BP is supported by previous studies. Higher intakes of dairy (≥ two serves/day) have been associated with lower BP in children [40,41,42], although regular and reduced fat dairy were not compared. A longitudinal study of adolescents found that increasing dairy intake was associated with decreases in BP for girls, particularly for cheese (generally a higher fat dairy product) [43]. Our results suggest the beneficial effects of dairy on BP in youth may not differ by fat content. In adults, reduced fat dairy may be more effective, but results are inconsistent [44]. Overall, there is a strong likelihood that dairy consumption benefits BP [45], potentially due to factors outside fat content. High protein, calcium and potassium, low sodium and bioactive lactotripeptides found in dairy may influence BP through a variety of mechanisms including via effects on nitric oxide, endothelial function, vasodilation, arterial stiffness, and water retention [44]. In our study, significant associations with BP were only seen in boys, which may suggest a gender-related increased sensitivity to these effects or the existence of a threshold, given that intake of dairy was higher in boys.

We found three published studies that have compared regular and reduced fat dairy intake with MetS or metabolic related outcomes in adolescence: Te Velde *et al*. followed Dutch adolescents to adulthood [46]; Malik *et al*. [47], retrospectively examined adolescent intakes recalled from high school years and compared them with adult outcomes in The Nurses’ Health Study II, and Mohammadi *et al*., conducted cross-sectional investigations of Iranian adolescents [48]. Te Velde *et al*., showed that participants with at least two MetS risk factors at 36 years of age had consumed more reduced fat and less regular fat dairy at 21 years, but observed no significant associations with earlier adolescent intake [46]. The study did not examine males and females separately, likely due to sample size limitations. In The Nurses’ Health Study II, consumption of regular but not reduced fat dairy products in adolescence was inversely associated with later development of type 2 diabetes, although both low and regular fat consumption were inversely associated when the women were examined cross-sectionally as middle aged adults [47]. Mohammadi *et al*. [48] found no significant associations with MetS for either reduced fat or regular fat dairy, with girls and boys grouped together for analysis.

Similar results were found in a large multi-cohort study of US adults, in which neither reduced fat or regular fat dairy intake was significantly associated with risk of type 2 diabetes [49]. Meta-analyses in adults also found that intake of regular fat dairy was not significantly associated with increased risk of type 2 diabetes [50], hypertension [51,52] or cardiovascular disease [53], although reduced fat dairy was found to be significantly inversely associated with risk in each of these studies. Intake of cheese, despite being a higher fat dairy product, was also found to be significantly inversely associated with risk in some of these meta-analyses [50,53].

Taken together with our findings, existing observational evidence suggests that intake of regular fat dairy is not associated with increased metabolic or cardiovascular risk. However, these studies do not represent cause and effect. Some people at higher risk of poor health outcomes may have chosen reduced fat dairy products specifically due to their risk status. Randomized trials can address this limitation. Benatar *et al*. [54] conducted a meta-analysis of randomized trials in adults that increased dairy intake using either reduced fat or regular fat products for at least one month. Overall, results were similar between reduced and regular fat products, with modest weight gain and with no or minor effects on other cardiometabolic risk factors. However, none of the trials in this meta-analysis directly compared the effects of reduced fat with regular fat dairy.

In our study, increasing intake of reduced fat but not regular fat dairy showed a small association with lower HDL-cholesterol and a higher total: HDL-cholesterol ratio in boys. Along the same lines, low but not regular fat dairy was associated with a lower total: HDL-cholesterol ratio in girls; however, this association was not significant after full adjustment. As higher HDL-cholesterol values are associated with improved cardiovascular risk [55], this finding suggests that regular fat dairy is associated with a slightly more beneficial lipid profile. This association may be due to additional fatty acids provided by regular fat dairy products improving HDL-cholesterol. Saturated fat can increase total and LDL-cholesterol, but it may also increase HDL-cholesterol [56]. Lower fat dairy intakes may therefore result in marginally lower HDL-cholesterol along with LDL-cholesterol. Short term diets high in regular fat milk and butter have been shown to increase both LDL- and HDL-cholesterol, with minimal change or even improvement to the total: HDL-cholesterol ratio [57]. Higher HDL-cholesterol has numerous beneficial functions within the cardiovascular system, including removal of cholesterol from blood vessel walls, assisting in blood vessel dilation and reducing injury to blood vessels [58].

Changing the type of dairy product consumed is likely to have flow on effects to other parts of the diet. A randomized controlled trial investigating changing children’s consumption from regular fat to reduced fat dairy foods found that although saturated fat intake was reduced, overall energy intake remained similar, suggesting the energy from saturated fat was replaced by other sources [59]. Resulting health effects are likely to depend on what replaces saturated fat—polyunsaturated fat, but not carbohydrate, has been associated with a reduction in coronary heart disease [60]. Given that research suggests energy intakes tend to remain similar in children, consumption of regular fat dairy products in place of reduced fat products may potentially decrease the amount of refined carbohydrate snack foods eaten by children and adolescents. Intakes of these foods are likely to be high in some adolescents, with an Australian study reporting that over forty per cent of adolescent daily food energy comes from confectionery, biscuits, cakes and other products which fall outside the core groups of fruit, vegetables, meat and alternatives, dairy and grains [61].

It is also important to remember that people consume whole foods rather than separate nutrients. Foods containing saturated fat may have varying effects on health due to other nutrients or bioactive properties in the food, as well as different types of saturated fats present. For example, margaric acid (17.0), a saturated fatty acid moderately correlated with dairy fat consumption, has been significantly associated with a lower risk of coronary disease [62]. Thus, regular fat dairy products may have different effects on health compared with other high saturated fat foods [9]. In addition, the fat composition of the dairy products themselves may differ by farming practices, with fatty acid content dependent on the cows’ feed. Milk from cows that are predominantly pasture fed, as in Australia, is likely to have a more beneficial fatty acid profile than milk from grain fed cows [13].

As recommended by some statisticians [63,64], no mathematical correction was made for testing multiple associations, instead all results are reported. Thus, some of our observed significant associations may be due to chance. Although the Raine Study is a population-based cohort, not all subjects completed every aspect of the adolescent follow-ups, and those completing dietary assessments tended to have relatively lower body mass indices, older mothers and higher family income [21,38,39]. This therefore limits the generalization of our results to the wider adolescent population. Although the FFQ we used was tailored for, and tested in, our adolescent population, it is only an estimate of intake, given the limitations of self-reporting. To standardize serve sizes, we used calcium equivalents but this has limitations when analyzing different dairy products, as they cover such a wide range of nutrient profiles. Dairy foods with lower calcium levels relative to other nutrients would be potentially underrepresented as a result. A further limitation of this study is that BP values obtained from sitting and supine positions cannot be assumed to be equivalent, as supine values tend to be slightly lower [65]. This may mean that the association observed with dairy and BP is understated, as smaller differences are harder to detect.

An important strength of our study was the ability to adjust for evaluated marks of overall diet quality [21], as well as energy intake and infant feeding. Other strengths of our study include its prospective design, the population based sample and use of comprehensive metabolic measures.

Our study of adolescents found that associations between dairy intake and cardiometabolic risk factors were similar for regular and reduced fat products. Potentially, either reduced fat or regular fat dairy products may be suitable for healthy development in this age range, and good quality dietary trials are required in this area. Given that intakes of dairy can often be low in adolescents, advocating a choice of either regular or reduced fat products may encourage more adolescents to meet recommended dairy intakes. However, determination of the optimal number of dairy serves also requires further investigation [66]. We aim to continue our prospective analyses of associations with cardiometabolic factors as participants in the Raine Cohort enter adulthood. Our interesting findings with HDL-cholesterol support future development of randomised dietary trials to determine whether a change to regular fat dairy could potentially benefit adolescent cardiometabolic health.

## Abbreviations

Body mass index = BMI, confidence interval = CI, Commonwealth Scientific and Industrial Research Organization = CSIRO, food frequency questionnaire = FFQ, generalized estimating equations = GEE, high-density lipoprotein = HDL, homeostasis model assessment for insulin resistance = HOMA-IR, International Diabetes Federation = IDF, low-density lipoprotein = LDL, metabolic syndrome = MetS, odds ratio = OR, television = TV.

## References

[1] National Health and Medical Research Council (2013). Australian Dietary Guidelines: Summary.

[2] Health Canada (2011). Canada’s Food Guide.

[3] U.S. Department of Agriculture, U.S. Department of Health and Human Services (2010). Dietary Guidelines for Americans.

[4] Ludwig D.S., Willett W. (2013). Three daily servings of reduced-fat milk: An evidence-based recommendation?. JAMA Pediatr..

[5] Louie J.C.Y., Flood V.M., Hector D.J., Rangan A.M., Gill T.P. (2011). Dairy consumption and overweight and obesity: A systematic review of prospective cohort studies. Obes. Rev..

[6] Holmberg S., Thelin A. (2013). High dairy fat intake related to less central obesity: A male cohort study with 12 years’ follow-up. Scand J Prim Hlth Care.

[7] Eriksson S., Strandvik B. (2010). Food choice is reflected in serum markers and anthropometric measures in healthy 8-year-olds. Eur. e-J. Clin. Nutr. Metab..

[8] Noel S.E., Ness A.R., Northstone K., Emmett P., Newby P.K. (2011). Milk intakes are not associated with percent body fat in children from ages 10 to 13 years. J. Nutr..

[9] O’Sullivan T.A., Hafekost K., Mitrou F., Lawrence D. (2013). Food sources of saturated fat and the association with mortality: A meta-analysis. Am. J. Public Health.

[10] Soedamah-Muthu S.S., Ding E.L., Al-Delaimy W.K., Hu F.B., Engberink M.F., Willett W.C., Geleijnse J.M. (2011). Milk and dairy consumption and incidence of cardiovascular diseases and all-cause mortality: Dose-response meta-analysis of prospective cohort studies. Am. J. Clin. Nutr..

[11] Goldbohm R.A., Chorus A.M.J., Garre F.G., Schouten L.J., van den Brandt P.A. (2011). Dairy consumption and 10-y total and cardiovascular mortality: A prospective cohort study in the Netherlands. Am. J. Clin. Nutr..

[12] Azadbakht L., Mirmiran P., Esmaillzadeh A., Azizi F. (2005). Dairy consumption is inversely associated with the prevalence of the metabolic syndrome in Tehranian adults. Am. J. Clin. Nutr..

[13] Kratz M., Baars T., Guyenet S. (2013). The relationship between high-fat dairy consumption and obesity, cardiovascular, and metabolic disease. Eur. J. Nutr..

[14] Huang T.T.K., McCrory M.A. (2005). Dairy intake, obesity, and metabolic health in children and adolescents: Knowledge and gaps. Nutr. Rev..

[15] Parker C., Vivian W., Oddy W., Beilin L., Mori T., O’Sullivan T.A. (2012). Changes in dairy food and nutrient intakes in Australian adolescents. Nutrients.

[16] Baird D.L., Syrette J., Hendrie G.A., Riley M.D., Bowen J., Noakes M. (2012). Dairy food intake of Australian children and adolescents 2–16 years of age: 2007 Australian National Children’s Nutrition and Physical Activity Survey. Public Health Nutr..

[17] Newnham J.P., Evans S.F., Michael C.A., Stanley F.J., Landau L.I. (1993). Effects of frequent ultrasound during pregnancy: A randomised controlled trial. Lancet.

[18] Baghurst K.I., Record S.J. (1984). A computerised dietary analysis system for use with diet diaries or food frequency questionnaires. Community Health Stud..

[19] Ambrosini G.L., Oddy W.H., Robinson M., O’Sullivan T.A., Hands B.P., de Klerk N.H., Silburn S.R., Zubrick S.R., Kendall G.E., Stanley F.J. (2009). Adolescent dietary patterns are associated with lifestyle and family psycho-social factors. Public Health Nutr..

[20] Food Standards Australia New Zealand Nutrient Tables for Use in Australia. http://www.foodstandards.gov.au/monitoringandsurveillance/nuttab2006/onlineversionintroduction/onlineversion.cfm.

[21] Ambrosini G.L., de Klerk N.H., O’Sullivan T.A., Beilin L.J., Oddy W.H. (2009). The reliability of a food frequency questionnaire for use among adolescents. Eur. J. Clin. Nutr..

[22] National Health and Medical Research Council (2013). Australian Dietary Guidelines.

[23] Baldwin E.M., Barrowman E.M., Cameron S., McDonnell E.D., Russel S.M., Williams W.I., The Trustees of the Home Economics Teachers Group (1967). Cookery—The Australian Way.

[24] Taste Australia. http://www.taste.com.au.

[25] Bonora E., Targher G., Alberiche M., Bonadonna R., Saggiani F., Zenere M., Monauni T., Muggeo M. (2000). Homeostasis model assessment closely mirrors the glucose clamp technique in the assessment of insulin sensitivity: Studies in subjects with various degrees of glucose tolerance and insulin sensitivity. Diabetes Care.

[26] Matthews D., Hosker J., Rudenski A., Naylor B., Treacher D., Turner R.C. (1985). Homeostasis model assessment: Insulin resistance and beta cell function from fasting plasma glucose and insulin concentrations in man. Diabetologia.

[27] Jolliffe C.J., Janssen I. (2007). Development of age-specific adolescent metabolic syndrome criteria that are linked to the Adult Treatment Panel III and International Diabetes Federation criteria. J. Am. Coll. Cardiol..

[28] Huang R.-C., Mori T.A., Burke V., Newnham J., Stanley F.J., Landau L.I., Kendall G.E., Oddy W.H., Beilin L.J. (2009). Synergy between adiposity, insulin resistance, metabolic risk factors, and inflammation in adolescents. Diabetes Care.

[29] Ambrosini G.L., Oddy W.H., Huang R.C., Mori T.A., Beilin L.J., Jebb S.A. (2013). Prospective associations between sugar-sweetened beverage intakes and cardiometabolic risk factors in adolescents. Am. J. Clin. Nutr..

[30] Appannah G., Pot G.K., Huang R.C., Oddy W.H., Beilin L.J., Mori T.A., Jebb S.A., Ambrosini G.L. (2015). Identification of a dietary pattern associated with greater cardiometabolic risk in adolescence. Nutr. Metab. Cardiovasc. Dis..

[31] Steinberger J., Daniels S.R., Eckel R.H., Hayman L., Lustig R.H., McCrindle B., Mietus-Snyder M.L. (2009). American Heart Association Atherosclerosis, Hypertension, and Obesity in the Young Committee of the Council on Cardiovascular Disease in the Young; Council on Cardiovascular Nursing; and Council on Nutrition, Physical Activity, and Metabolism. Progress and Challenges in Metabolic Syndrome in Children and Adolescents: A Scientific Statement From the American Heart Association. Circulation.

[32] Huang R.-C., Mori T.A., Burrows S., Le Ha C., Oddy W.H., Herbison C., Hands B.H., Beilin L.J. (2012). Sex dimorphism in the relation between early adiposity and cardiometabolic risk in adolescents. J. Clin. Endocrinol. Metab..

[33] Savva S.C., Tornaritis M., Savva M.E., Kourides Y., Panagi A., Silikiotou N., Georgiou C., Kafatos A. (2000). Waist circumference and waist-to-height ratio are better predictors of cardiovascular disease risk factors in children than body mass index. Int. J. Obes. Relat. Metab. Disord..

[34] Boreham C.A., Paliczka V.J., Nichols A.K. (1990). A comparison of the PWC170 and 20-MST tests of aerobic fitness in adolescent schoolchildren. J. Sports Med. Phys. Fit..

[35] Rockett H.R.H., Breitenbach M., Frazier A.L., Witschi J., Wolf A.M., Field A.E., Colditz G.A. (1997). Validation of a youth/adolescent food frequency questionnaire. Prev. Med..

[36] Hanley J.A., Negassa A., Edwardes M.D., Forrester J.E. (2003). Statistical analysis of correlated data using generalized estimating equations: An orientation. Am. J. Epidemiol..

[37] IBM Corp. (2012). IBM SPSS Statistics for Windows.

[38] O’Sullivan T.A., Ambrosini G.L., Beilin L.J., Mori T.A., Oddy W.H. (2011). Dietary intake and food sources of fatty acids in Australian adolescents. Nutrition.

[39] O’Sullivan T.A., Lyons-Wall P., Bremner A.P., Ambrosini G.L., Huang R.C., Beilin L.J., Mori T.A., Blair E., Oddy W.H. (2010). Dietary glycaemic carbohydrate in relation to the metabolic syndrome in adolescents: Comparison of different metabolic syndrome definitions. Diabet. Med..

[40] Yuan W.L., Kakinami L., Gray-Donald K., Czernichow S., Lambert M., Paradis G. (2013). Influence of dairy product consumption on children’s blood pressure: Results from the quality cohort. J. Acad. Nutr. Diet.

[41] Moore L.L., Singer M.R., Bradlee M.L., Djousse L., Proctor M.H., Cupples L.A., Ellison R.C. (2005). Intake of fruits, vegetables, and dairy products in early childhood and subsequent blood pressure change. Epidemiology.

[42] Rangan A.M., Flood V.L., Denyer G., Ayer J.G., Webb K.L., Marks G.B., Celermajer D.S., Gill T.P. (2012). The effect of dairy consumption on blood pressure in mid-childhood: CAPS cohort study. Eur. J. Clin. Nutr..

[43] Gopinath B., Flood V.M., Burlutsky G., Louie J.C.Y., Baur L.A., Mitchell P. (2014). Dairy food consumption, blood pressure and retinal microcirculation in adolescents. Nutr. Metab. Cardiovasc. Dis..

[44] McGrane M.M., Essery E., Obbagy J., Lyon J., MacNeil P., Spahn J., van Horn L. (2011). Dairy consumption, blood pressure, and risk of hypertension: An evidence-based review of recent literature. Curr. Cardiovasc. Risk Rep..

[45] Park K.M., Cifelli C.J. (2013). Dairy and blood pressure: A fresh look at the evidence. Nutr. Rev..

[46] Te Velde S.J., Snijder M.B., van Dijk A.E., Brug J., Koppes L.L., van Mechelen W., Twisk J.W. (2011). Dairy intake from adolescence into adulthood is not associated with being overweight and metabolic syndrome in adulthood: The Amsterdam Growth and Health Longitudinal Study. J. Hum. Nutr. Diet.

[47] Malik V.S., Sun Q., van Dam R.M., Rimm E.B., Willett W.C., Rosner B., Hu F.B. (2011). Adolescent dairy product consumption and risk of type 2 diabetes in middle-aged women. Am. J. Clin. Nutr..

[48] Ghotboddin Mohammadi S., Mirmiran P., Bahadoran Z., Mehrabi Y., Azizi F. (2015). The Association of Dairy Intake With Metabolic Syndrome and Its Components in Adolescents: Tehran Lipid and Glucose Study. Int. J. Endocrinol. Metab..

[49] Chen M., Sun Q., Giovannucci E., Mozaffarian D., Manson J., Willett W.C., Hu F.B. (2014). Dairy consumption and risk of type 2 diabetes: 3 cohorts of US adults and an updated meta-analysis. BMC Med..

[50] Aune D., Norat T., Romundstad P., Vatten L.J. (2013). Dairy products and the risk of type 2 diabetes: A systematic review and dose-response meta-analysis of cohort studies. Am. J. Clin. Nutr..

[51] Soedamah-Muthu S.S., Verberne L.D.M., Ding E.L., Engberink M.F., Geleijnse J.M. (2012). Dairy Consumption and Incidence of Hypertension: A Dose-Response Meta-Analysis of Prospective Cohort Studies. Hypertension.

[52] Ralston R.A., Lee J.H., Truby H., Palermo C.E., Walker K.Z. (2012). A systematic review and meta-analysis of elevated blood pressure and consumption of dairy foods. J. Hum. Hypertens..

[53] Qin L.-Q., Xu J.-Y., Han S.-F., Zhang Z.-L., Zhao Y.-Y., Szeto I.M. (2015). Dairy consumption and risk of cardiovascular disease: An updated meta-analysis of prospective cohort studies. Asia Pac. J. Clin. Nutr..

[54] Benatar J.R., Sidhu K., Stewart R.A. (2013). Effects of high and low fat dairy food on cardio-metabolic risk factors: A meta-analysis of randomized studies. PLoS ONE.

[55] Boden W.E. (2000). High-density lipoprotein cholesterol as an independent risk factor in cardiovascular disease: Assessing the data from Framingham to the Veterans Affairs High--Density Lipoprotein Intervention Trial. Am. J. Cardiol..

[56] Micha R., Mozaffarian D. (2010). Saturated fat and cardiometabolic risk factors, coronary heart disease, stroke, and diabetes: A fresh look at the evidence. Lipids.

[57] Huth P.J., Park K.M. (2012). Influence of dairy product and milk fat consumption on cardiovascular disease risk: A review of the evidence. Adv. Nutr. Int. Rev. J..

[58] Scharf R.J., Demmer R.T., DeBoer M.D. (2013). Longitudinal evaluation of milk type consumed and weight status in preschoolers. Arch. Dis. Child..

[59] Hendrie G.A., Golley R.K. (2011). Changing from regular-fat to low-fat dairy foods reduces saturated fat intake but not energy intake in 4–13-year-old children. Am. J. Clin. Nutr..

[60] Jakobsen M.U., O’Reilly E.J., Heitmann B.L., Pereira M.A., Bälter K., Fraser G.E., Goldbourt U., Hallmans G., Knekt P., Liu S. (2009). Major types of dietary fat and risk of coronary heart disease: A pooled analysis of 11 cohort studies. Am. J. Clin. Nutr..

[61] Golley R.K., Hendrie G.A. (2012). The impact of replacing regular- with reduced-fat dairy foods on children’s wider food intake: Secondary analysis of a cluster RCT. Eur. J. Clin. Nutr..

[62] Chowdhury R., Warnakula S., Kunutsor S., Crowe F., Ward H.A., Johnson L., Franco O.H., Butterworth A.S., Forouhi N.G., Thompson S.G. (2014). Association of dietary, circulating, and supplement fatty acids with coronary risk: A systematic review and meta-analysis. Ann. Int. Med..

[63] Rothman K.J. (1990). No adjustments are needed for multiple comparisons. Epidemiology.

[64] Saville D.J. (1990). Multiple comparison procedures: the practical solution. Am Stat.

[65] Netea R., Smits P., Lenders J., Thien T. (1998). Does it matter whether blood pressure measurements are taken with subjects sitting or supine?. J. Hypertens..

[66] Weaver C.M. (2014). How sound is the science behind the dietary recommendations for dairy?. Am. J. Clin. Nutr..

